# Annotations

**Published:** 1886-11-27

**Authors:** 


### Annotations.

At many a railway station the hurrying passenger casts a glance on the big brown box with its wide-open mouth, which tells him that "newspapers and periodicals will be thankfully received for the —— Hospital." He has not time at the moment to offer a mouthful to the suppliant box, but he thinks he will remember it when he gets home. Does he remember it? Sometimes; but only sometimes. If all of us carried out our good intentions, the chaplain to the City of London Chest Hospital, Rev. H. Constable, M.A., would not need to write to the daily papers, asking for contributions of literature of this kind. What a pity it is that so few of us are as good as our impulses! A mad patient, in a recent number of The Hospital, gave it as his opinion that the real explanation of the origin of evil was "bad handwriting; if everybody would only write a good hand there would be no evil." Your contributor, who humbly hopes that he is still sane, ventures to suggest that perhaps the origin of evil was in the failure of people to carry out their own good intentions. At any rate, if that were not the real origin of evil, it undoubtedly does originate much evil; and, among other things, it does not send its old newspapers and periodicals to the nearest hospital. "Every schoolboy knows" that the way to a certain place is paved with good intentions. We should like all the paving-stones on that particular road to be torn up, so that the road itself might become absolutely impassable. But, at all events, let no new stones be laid upon it by the friends of hospitals; but let their intentions to give donations, subscriptions, flowers, fruits, periodicals, and newspapers, all be carried out as soon as formed, and in the fullest and completest possible way.

**Books and Periodicals.**

The editor of the *Wolverhampton Chronicle* complains that at the Guest Hospital, Dudley, "no facilities are afforded the representatives of the press for the obtaining of information. When a reporter calls the porter knows nothing, but refers the reporter to the nurses, who in their turn decline to give any information without the sanction of the resident surgeon. As, however, the representatives of the press cannot always remain to see that official, they have either to go away without the desired information, or to get it as they can outside." We do not suppose there is any decided intention to withhold information, but merely a wish to avoid trouble. But hospital officials should remember that they are parts of a great system of things, and not isolated persons in isolated institutions; and that inasmuch as the system is maintained by the public, so the public has a natural desire and a right to know how affairs are conducted. Every hospital should provide in its regulations for the due supply of information to the accredited representatives of the press. It should not be in the power of careless or indifferent officials to prevent the gaining of information when applied for according to established rule. Hospitals, when properly conducted, have everything to gain, and nothing to lose, by the fullest publicity. We are glad to think that, as a rule, both the responsible managers and the regular officials afford all needed facilities to those who have an admitted right to ask for them.

**Who is Responsible?**

There used to be many strange anomalies to be met with in most hospitals. Of late these have very greatly diminished in number, but a few are still extant. The out-patients' department at the Lock Hospital in Dean Street, Soho, is well worthy of the attention of the "Man in the Crowd" who writes so eloquently in the daily press about Londoners and their habits. Here may, or at any rate recently could, be found out-patients in the women's department who have been in attendance for years and years. One woman has been an out-patient at this hospital for eight years and a half without any appreciable break in her attendance. Another has been five years, and altogether thirty-eight were found to have been under treatment for a period of upwards of two years. Such a state of affairs speaks eloquently of the want of system and management in the institution concerned, and should lead the governors to appoint a small and representative committee to inquire into the matter, with a view to its speedy improvement and reform.

**Eight Years an Out-patient.**

The case of Tanfani *v.* Spurgin, which was recently decided, is probably not yet terminated. Civilisation develops many complexities and terrors for those who enjoy its not wholly unmixed blessings. Medical men who pride themselves upon being near the apex of the pyramid of modern rationalism occupy the position of newly-whitewashed targets, and every man or woman who can command a rifle, or even a bow and arrows, thinks himself entitled to take aim at them. Generally the judges are approving parties, and by their behaviour to the doctors, and their usual summing up, indicate that it gives them a good deal of satisfaction to see a poor unfortunate medico "pilled" without mercy. Mr. Justice Denman has seen fit to take a different view, and his decision has made not only Dr. Spurgin, but the whole profession, breathe a little more freely. Every calling probably has its bugbear, and the bugbear of medicine is lunacy. One cannot help asking the question, "Why do doctors endure this state of things?" If they have not mental capacity enough to suggest new methods of procedure, they ought at least to have sufficient energy of just anger to put an end to the present intolerable position. As matters now stand, every medical man who signs a certificate of lunacy may, by that very signature, be

**Lunatics and Doctors.**

precipitating his own financial and professional ruin. The whole case is briefly this: there are a certain number of lunatics who, for their own welfare, and that of others, must be placed under restraint. Who is to do it? Are the judges the proper persons? And if not the judges, are the juries? But if neither judge nor jury be competent, who shall perform the duties? Where is the wisdom of the century concentrated in the greatest force? Is it in the Lords or the Commons, or among the Socialists, or the Home Rulers, or the Hall of Science philosophers? Perhaps a congress of parish clerks, or a conference of automatic chess-players might solve the problem. In any case, here is the difficulty, and somebody must deal with it. For want of a body of infallibles who will really do it perfectly, the general sense of the public will probably say that, on the whole, the doctors are the best persons to deal with the question. If so, then the least the public can do is to afford reasonable protection to the doctors. If that be not secured, all medical men who have a spark of British spirit in them will absolutely refuse to sign lunacy certificates on any terms whatever.

**Take care of your False Teeth.** Dr. B. was returning from a holiday in Scotland a few weeks ago, when it was his fortune to witness a very amusing incident, which was not without its pathetic side also. A pleasant-looking matron, short of fifty, was travelling south. She was dressed with unusual neatness, and was evidently in the best of spirits. In the course of conversation, the pleased dame informed the doctor that she was going all the way to a certain southern seaport to meet her husband, who had been across the seas for a long period. Tempted by the brightness of the weather, she put her head out of the window for a "breath of fresh air." The train was in full motion, and a strong wind blowing at the moment caused her to sneeze violently, when, to her dismay, out fell a whole mouthful of pearly-white artificial teeth. When she popped her head out of window she was forty-five: when she brought it in again she was sixty. Words cannot picture her trouble and distress. "How can I meet my husband after all this time without a tooth in my mouth? I thought he would be so delighted to see me, and now I can't look him in the face." She was inconsolable. *Moral*: If you have false teeth don't sneeze over a deep well or out of a railway carriage window.

---

Jenner's Statue.—Two interesting youths were looking one day at Dr. Jenner's statue in Kensington Gardens. Said one of the youths, "Do you know, Fred, there's a peculiar thing about this statue?" "No," said his friend, "I didn't; what's the matter with it?" "Why," said the other, "when it rains, it gets ever so much less; in fact, it *jenner*-ally becomes a mere *statue-wet*." Fred subsided.

